# A Policy Guide on Integrated Care (PGIC): Lessons Learned from EU Project INTEGRATE and Beyond

**DOI:** 10.5334/ijic.3295

**Published:** 2017-09-25

**Authors:** Liesbeth Borgermans, Dirk Devroey

**Affiliations:** 1 Department of Family Medicine and Chronic Care, Faculty of Medicine and Pharmacy, Vrije Universiteit Brussel, Pleinlaan 2, Brussels, BE

**Keywords:** integrated care, health policies, perspectives

## Abstract

Efforts are underway in many European countries to channel efforts into creating improved integrated health and social care services. But most countries lack a strategic plan that is sustainable over time, and that reflects a comprehensive systems perspective. The Policy Guide on Integrated Care (PGIC) as presented in this paper resulted from experiences with the EU Project INTEGRATE and our own work with healthcare reform for patients with chronic conditions at the national and international level. This project is one of the largest EU funded projects on Integrated Care, conducted over a four-year period (2012–2016) and included partners from nine European countries. Project Integrate aimed to gain insights into the leadership, management and delivery of integrated care to support European care systems to respond to the challenges of ageing populations and the rise of people living with long-term conditions. The objective of this paper is to describe the PGIC as both a tool and a reasoning flow that aims at supporting policy makers at the national and international level with the development and implementation of integrated care. Any Policy Guide on Integrated should build upon three building blocks, being a mission, vision and a strategy that aim at capturing the large amount of factors that directly or indirectly influence the successful development of integrated care.

## Introduction

The interest of policy makers in integrated care has been steadily growing over the last decade as a solution to tackle health challenges such as the increase in chronic diseases, multi-morbidity, the ageing of the population and the constrained use of resources. In most EU countries, funding is allocated to individual service providers and institutions rather than networks of organisations with shared goals. While integrated care was initially defined as ‘improved connectivity between different activities of the health system in order to provide better quality health services to users’, there is nowadays an increasing complexity attributed to its concept. The constructs commonly described in scoping literature include patient-centered care, care coordination, continuity of care, chronic disease management and integrated healthcare delivery [1,2,3,4,5,6,7,8,9,10,11,12,13,14,15,16,17,18]. This has resulted in growing confusion over its tangible and non-tangible components, and consequently its outcomes.

Policy makers who want to launch themselves into the development and implementation of strategic reform in favour of the development of integrated health and social care are consequently looking for answers on how to master the complexity of the integrated care. Any such attempt calls for a comprehensive approach and system-perspective with an emphasis on a life course approach to care and thus prevention, effective management of multi-morbid conditions and multidisciplinary approaches to care. Engagement of patients, families and communities is an essential component if innovation and effective care is to be realized.

With the aim to support national and international policy makers in their ambitions to develop integrated care, we present a Policy Guide on Integrated Care (PGIC) that builds on both findings from the EU FP-7 Project INTEGRATE (www.projectintegrate.eu) and our own work with healthcare reform for patients with chronic conditions at the national and international level [19,20,21]. The methods that have provided the basis to the development of the PGIC have been explained in large detail in a first paper in this series [22]. In short: six different sources were consulted. The first source were findings and recommendations from the different work packages of the EU Project INTEGRATE. A second source was a literature review on integrated care policies for people with chronic conditions. Other additional sources used were a) existing frameworks on chronic and people-centred/integrated care, b) key findings from other EU Projects targeting chronic illnesses/integrated care and c) a selected set of ‘best practices’ on integrated care from different countries and d) our own experiences with research and policy making in integrated care at the national and international level.

The starting point to the development of the PGIC is what patients with chronic conditions expect from any provider in the health care system, and what we think can be summarized as ‘compassionate and competent care’. Compassionate and competent care is essentially integrated, people-centered and values a bio-psycho-social approach to care emphasizing the importance of equity, and high-quality interventions across the life course and the entire health continuum and aims at better health outcomes, care experiences, and with a more efficient use of resources. The ultimate goal of the PGIC is to enable compassionate and competent care delivery. The PGIC builds upon three straightforward building blocks, being a mission, vision and a strategy that aim at capturing the large amount of factors that directly or indirectly influence the successful development of integrated care. Each of these building blocks and their respective components will be further outlined below.

## The Policy Guide on Integrated Care (PGIC)

### PGIC Building block 1: The mission

The Triple Aim framework serves as the foundation for organizations and communities to successfully navigate the transition from a focus on health care to optimizing health for individuals and populations [23]. This framework put forward the simultaneous pursuit of three aims: improving the experience of care, improving the health of populations, and reducing per capita costs of health care. No single actor alone has the capability to successfully pursue improving the health of a population since the Triple Aim explicitly requires health care organizations, public health departments, social service entities, school systems and employers to cooperate [24]. Fostering this cooperation requires an integrator that accepts responsibility for achieving the Triple Aim for the population [24]. Policy makers can act as integrators by making the right investments and creating a cogent set of high-level measures to monitor progress. The three dimensions of the Triple Aim, taken together, provide a useful framework for measuring value in health care. Value can be conceptualized as the optimization of the Triple Aim, recognizing that different stakeholders may give different weights to the three dimensions.

### PGIC Building block 2: The vision

When policy makers develop a vision on integrated care, three different components are considered important. *First*, they should profoundly understand the context for change. *Second*, they must understand the core principles that underpin integrated care. *Third*, they must understand the problems and societal challenges that can be addressed with integrated care.

#### Part 1 of the vision: Understanding the context for change

A first part of the vision includes the understanding of the context for change that involves patients (people) and their families, the health workforce, health managers, insurers and policy makers.

##### Change for people

The quality of care individuals of every generation seek is increasing. Widespread access to digital information due to new technology and greater personal expenditure on healthcare have both raised people’s expectations [25]. Shaping policy to foster integrated care will essentially start with a better understanding of the diverse needs of people with chronic conditions. Health needs should be the starting point of any transformation [26]. A growing number of people want more options and information about the care they receive, more input into decisions about their care and higher standards of treatment [25,27]. People don’t want to be medicalised either, and they want to be treated humanely—to be respected as individuals and dealt with equitably and compassionately [28]. People want having a comprehensive assessment with less redundancy, support for transitions into and out of the hospital as well as greater attention to mental health over the course of their disease process. On the staffing side, quicker response times, ongoing patient–provider communication and consistency between providers and across care units are considered important [29]. All the aforementioned elements drive the need for change from the patient, family and citizen perspective.

But we may not forget that what many people want is just the obvious – delivering what is needed and promised in a joined up and reliable fashion. In this sense, health care providers cannot blame the client for ‘being more demanding’ in a reluctance to move away from the ‘object of care’ mentality.

##### Change for the health workforce

Professional caregivers who deal with complex and multiple health problems in their patients can no longer rely on traditional approaches to care which focuses on individual diseases. Integrated care requires a return to core Hippocratic principles [30]. New paradigms of care delivery are needed with the shift to population health management and proactive care to be essential. But current guidelines are not designed to consider the cumulative impact of treatment recommendations on people with several conditions, nor to allow comparison of relative benefits or risks [31]. In people with multimorbidity current guideline recommendations rapidly cumulate to drive polypharmacy, without providing guidance on how best to prioritise recommendations for individuals in whom treatment burden will sometimes be overwhelming. But multimorbidity alone cannot explain the complexity of care needs and further stratification of the general population based on care needs is necessary for allocating resources and developing personalized, and patient-centered care plans [32]. The notions of self-care, co-ownership of therapeutic options, discussing planned outcomes and informed expression of choice will require re-thinking of the professional identity of involved health care professionals. In addition, eHealth and mHealth solutions will become increasingly important for improving participatory continuity of care.

##### Change for health managers

Health managers that have succeeded in fostering integrated care have gone beyond mainstream frameworks for quality improvement based on clinical measurement and audit and have adopted a strategic organizational approach to patient focus [33]. Delivering individualised, integrated care entails dissolving ‘the classic divide between primary care and secondary care, between physical and mental health, between health and social care, between prevention and treatment’ and between private and public institutions. In the future, new paradigms of data custodianship will be needed in a complex multi-provider setting. The construct, legal mandate, resourcing, governance and audit need careful consideration, in order to ensure trust by all parties, as well as efficiency and reliability [34].

##### Change for insurers

Hospital payment models still largely make use of fee-for-service or case payments (e.g. diagnostic related groups) promoting volume with little consideration for quality of care. Primary and social care funding is often very distinct and differently sourced from that for hospital care despite the fact that these services support the same people and have key inter-dependencies [34]. Most existing payment models are creating barriers to innovation by rewarding volume, not value for the money spent. Moreover, insurers promote health services contractual arrangements with single providers perpetuating the “silo effect” and enhancing fragmentation of care, inhibiting the creation of innovative care delivery models that will likely find new ways of integrate care [35]. There are also perversities of some contracting regimens, which contract bundles of supportive care based on short-term (e.g. annual) cycles, jeopardizing care continuity, patient choice and patient-centered quality outcomes.

##### Change for policy makers

The current way of caring for people with chronic conditions is economically unsustainable because in most EU countries it is based on a costly, hospital-centred health system. In many countries, the workforce available to provide health and care services is static or diminishing, due to cost constraints on levels of professional staff and to limited numbers available to undertake lower-paid caring tasks [34]. Nowadays the ‘medical model’ is still the model for which virtually all of the resources are used. i.e., physicians, hospitals, nursing homes. In such model, interventions can quickly lose their connection with population health, and the wider determinants of health are often neglected [36]. Using the social determinants of health model (which is a more recent force), it is important to understand how education, wealth, and similar characteristics affect the health status of individuals and communities. To this complex scene must now be added revolutions in information and mobile technology and the unprecedented growth of research and applications in the area of nanoscience and nanotechnology. Other anticipated innovations in medicine relate to drug delivery, diagnostics, cell therapy and production of biocompatible materials.

#### Part 2 of the vision: Core principles that underpin integrated care

A second part of the vision is knowledge that policy must possess on the core principles that underpin integrated care. Effective health and care delivery must by definition focus on the individual, which means that ‘patient-centeredness’ is a core principle that underpins integrated care. Other important principles are: e.g. ‘led-by whole systems thinking’, ‘comprehensive’, ‘evidence-informed’, ‘co-produced’, ‘collaborative’, ‘empowering’, ‘engaging’, ‘respectful’, ‘endowed with rights and responsibilities’, ‘governed through shared accountability’, ‘goal-oriented’, ‘safe’, ‘effective’, ‘efficient’, ‘timely’, ‘sustainable’ and ‘equitable’ [7]. All these principles are drivers of excellent experience of care.

#### Part 3 of the vision: Understanding what problems can be addressed with integrated care

A third part of the vision is that policy makers must understand what problems can be addressed with integrated care, applying different perspectives. An overview of problems in care delivery for people with chronic conditions that can be addressed with integrated care is provided in Table 1.

**Table 1:** Overview of issues in chronic care delivery that can be addressed by integrated approaches to care organization and/or financing.

Perspectives	Issues in chronic care delivery that can be addressed by integrated approaches to care organization and/or financing

**Patient/carers perspective**	Services difficult to navigate, disempowering, burdensome Poor geographical access to care Poor patient-doctor communication Poor co-production (of services) Poor health literacy (knowledge on health & insufficient competencies on self-management) Poor peer support and the number of peer support programmes Poor patient education Insufficient compliance Insufficient use of information technologies Insufficient patient-reported outcome measures Insufficient support of carers
**Provider perspective**	Lack of centrality of client needs Disease-focused approaches Episodic medical orientation Wrong/inadequate services at the wrong time Fragmented chains of command Duplicated supervision Lack of bio-psycho-social integration of care at the individual level Lack on integration between health and social care Lack of co-ordination Medication errors Physician patient communication failure Poor doctor-patient communication Burnout in providers
**Health care manager and insurer perspective**	Avoidable hospitalisation Insufficient integration within primary care Insufficient integration between primary care and hospital care Insufficient integration between primary care and long-term care/palliative care Insufficient integration between medical and mental care Insufficient integration between health and social care Insufficient focus on prevention Fragmented and inadequate funding mechanisms Inadequate payment and rewarding systems
**Policy maker perspective**	Inadequate life-course approaches to care Inadequate Health in All Policies approaches Inadequate payment and rewarding systems No agreement on quality measures for integrated care Inadequate Information system systems Multiple transaction costs

### PGIC Building block 3: The strategy

A strategy on integrated care essentially includes a range of measures that can be implemented at the local health economy level, but with appropriate support and inclusion of directives developed at the national and international level. Policy makers should opt for a comprehensive and life course approach that is grounded in the right to health for every individual [37].

#### Entry points to integrated care strategies

Numerous entry points can be identified to the development of a strategy on integrated care for people with chronic conditions. Policy makers have the freedom to choose from the different entry points as presented in this paper, meaning there is no preferred chronological order. Any regulatory framework created by policy makers should allow for creativity and self-organisation from the bottom-up rather than prescribing a detailed blue print policy [38]. Policy makers can opt for both top-down and bottom-up strategies with integrative potential. Central to any strategy is the idea of value chains. What is crucial in these “chains” ideas is that each link in the chain adds up some value to the previous one.

#### Strategy Entry Point 1: Regulatory frameworks for collaborative entities and teams

A first potential entry point is the implementation of regulatory frameworks for collaborative entities and teams (coupled with financial incentives and/or changes in payment systems). The objectives of such regulatory frameworks are: improved 1. care co-ordination, 2. integration of medical and social/mental/community care, 3. inter-professional and inter-organisational governance, 4. relationship continuity with health professionals, 5. use of evidence-based medicine and 6. continuous discharge planning.

Collaborative entities and teams range from disease-based collaborative structures over Accountable Care Organizations (ACOs). Policy makers can opt for one overarching regulatory framework that applies to all types of collaborative entities and teams, or opt for more specific frameworks in support of different types of collaborative initiatives. It is key to any regulatory framework for collaborative entities and teams to include: a set of disease and non-disease specific integrated care indicators. No established set of indicators for measuring integrated care is currently available, and indicators used can be disease and/or non-disease specific. Overall, there is a strong need for international comparable integrated care indicators to highlight where significant variations between countries (or regions) exist, and to consequently call for their explanation and possible filling [39].

Provider payments should be strategized to encourage performance improvement to achieve the Triple Aim. Value-based payments reforms range from incremental approaches aimed at improving existing volume-based models using coordination and performance-based incentives or monitoring over bundled payment and retrospective/prospective full capitation models. Important aspects of payment reform design are alignment of the incentives with system goals, a strive for consistency in incentives and payment methods across providers/payers and to address provider protection from unavoidable risk as well as variation in patient morbidity.

#### Strategy Entry point 2: Regulatory frameworks for population health management

A second potential entry point is the development of regulatory frameworks for population health management. The objectives of regulatory frameworks for population health management are: Improving 1. outcomes of care, 2. experiences of care and 3. reduction of per capita cost. Population health management has made significant inroads due to the emergence of integrated care delivery systems. It is concerned with both the definition and measurement of health outcomes and the roles of determinants [48]. It also involves providing a wide spectrum of health care services that are directed at behavioral changes and encouraging healthy lifestyles to obtain optimal outcomes. Population health management implies the use of ‘Triple Aim’ indicators which proposes three linked goals — improving the individual experience of care, reducing per capita cost of care, and improving the health of populations.

**Table 2:** Evidence-based integrated care policies to the development of collaborative entities & teams.


***Examples:***
Integrated Care Certification (ICC) programmes Contracting with collaborative entities for services with explicit agreements about quality and equity Integrated Delivery Networks in primary care (community-based multidisciplinary teams) [19,40] Accountable Care Organizations (ACOs) [41] Integration of mental health and social services [42,43] Integration of mental and physical health care [19] Medical homes [28] Co-location policies in primary care Use of multidisciplinary guidelines, care plans and protocols [19,44] Handover strategies from hospital to primary care [45] Value-based financing/incentives targeting collaborative efforts & quality of care [46,47]


**Table 3:** Evidence-based integrated care policies to population health management.


***Examples:***
Define patient cohorts and prioritize them based on their relative importance to the health of the overall population to be managed [49] Population needs assessment [50] Multidimensional frailty assessment [51] Health registries [52] Risk stratification [53] Predictive analytics to model medical conditions to identify high-risk patients [54] Pooling of budgets between health care and social care [55]


**Table 4:** Evidence-based integrated care policies to the implementation of educational and professional reforms.


***Examples:***
Standardizing core competencies for coordinated/integrated health services delivery [62] Regulatory frameworks for professional accreditation (e.g. clinical licensing; certifications and periodic re-certification examinations for health professionals) [63] Strengthening regulators of education and services to ensure that services are up to a pre-determined standard [20] Legal changes (e.g. shift of competences of providers) [64] Promoting of particular medical specialities (e.g. family medicine, geriatrics and gerontology) Providing financial support for medical schools and residency programmes that adopt appropriate educational innovations (e.g. simulation methods, learning in the community, inter-professional education, admission procedures, faculty development) practices [65] Regulatory frameworks for human resources management [42] Increasing efforts at planning and forecasting [20] Registration of health professionals (e.g. ensuring that licences are up to date) [66] Enhancing mechanisms to voice patient needs (patient associations provide feedback on the health workforce performance, support the development of health professional curricula, set benchmarks and indicators of services) [67]


**Table 5:** Evidence-based integrated care policies to the development of (e)health literacy.


***Examples:***
Use of mass media campaigns on healthy lifestyles and certified health websites [71] Targeted educational packages and life style programmes Supported self-management (e.g. diabetes, obesity, cancer, asthma and heart failure) [72,73,74] Personalised care planning [75] Integrating and financing (nurse) educators, patient navigators [76,77], community health workers and case managers [78,79] in primary and secondary care practices and hospitals [80] Patient expert programmes facilitated by lay volunteers Community participation in planning and goal-setting (e.g. community consultations through committees and participatory groups) [81] Patient and service user groups (e.g. in the development of patient charters) Strategies that encourage lay, parental and family-led advice and support in local communities Shared decision making between people and health care professionals [82,83,84] Giving people access to personal health records [85] (Financial) incentives related to mutually defined health goals [86]


**Table 6:** Evidence-based integrated care policies to the prevention of ACEs.


***Examples:***
Strengthening (group-based) parenting skills to yield benefits in relation to physical and mental health (e.g. alleviating aspects of family adversity which may negatively affect parenting and delivery of parenting interventions) [96] Service design that recognizes the role and importance of schools in relation to children Preventive health services in public education [97] Use of prediction models for child maltreatment recurrence [98] Documentation of social determinants of health in child health services health records


#### Strategy Entry Point 3: Regulatory frameworks for educational and professional reforms

A third potential entry point is the implementation of new regulatory frameworks for educational and professional reforms for doctors, nurses, physiotherapists, occupational therapists, dieticians, social workers, amongst others. The objectives of policies on frameworks for educational and professional reforms are: 1. the development of new skill-sets/competencies for the entire health workforce, 2. improved inter-professional education and 3. improved staffing and task delegation within the health workforce. Meeting changing health care needs must begin at the foundation, in essence, in education [56]. Especially medical education still focuses on what a physician does in face-to-face contact with the patient [57]. Looking to the future, medical education should evolve to include preparation for (the ideal of) biopsychosocial chronic care [58] and a team approach to care [59] via practical training for multispecialty collaborative practice and preparing physicians to be leaders of primary care teams that include e.g. non-physician providers, cost-effective care in clinical practice, increased training in geriatrics, and “on ramps” and “off ramps” along the physician career path for flexible training over a lifetime [56]. A central feature of educational and professional reforms is the emphasis on patient-centered decision making (PCDM) which is the process of identifying clinically relevant, patient-specific circumstances and behaviors to formulate a contextually appropriate care plan [60,61]. Advanced Practitioner education and roles are important. There also need to be a mechanism of support to practitioners working solo in domiciliary settings- help with advice and reflective practice, but also a support mechanism in the event of putative adverse outcomes, where they may be landed with inappropriate blame.

#### Strategy Entry Point 4: A life course approach to the development of health literacy

A fourth potential entry point is a life course approach to the development of health literacy and e-literacy. Health literacy refers to people’s knowledge, motivation and competence to access, understand, appraise and apply health information in order to make judgments and take decisions about health care, disease prevention and health promotion to maintain or improve quality of life throughout their lives [68]. The objectives of policies to improve health literacy are: 1. empowerment of citizens, patients, family (caregivers) and communities through improved knowledge, self-management, self-identification, trust, authority to partner, self-efficacy, and co-production of services, 2. Improving access and navigation of the healthcare system, 3. Improving compliance to follow-up appointments, medication and instructions for at-home care, 4. Increased patient satisfaction and, 5. Lowering health care spending.

Health literacy underlines the importance of managing health, self-monitoring, communication with health professionals, and role and emotions related to chronic conditions. Low health literacy is linked to less use of preventive care, reduced safety of care due to medication errors and poor adherence to medication and treatment, more hospitalisation, worse health outcomes and greater risk of death [69]. Health literacy concepts are now being established, but need to be bedded into e-literacy as a specific area of citizen activity [34]. Because patients and families are diverse in their desire and ability to engage, it is important to consider how we can tailor efforts to meet patients and families where they are, address specific needs and concerns, and best facilitate their engagement [70].

#### Strategy Entry Point 5: Preventing childhood adverse experiences

A fifth potential entry point to the promotion of integrated care, and especially the prevention of chronic diseases, is the prevention of childhood adverse experiences. The objectives of policies to prevent ACEs are: 1. prevention of chronic illnesses and risk-behaviors, 2. improved quality of life of citizens, and 3. cost reduction. There is compelling evidence that different types of trauma in early life are important risk factors for poor health in adulthood, including autoimmune and other chronic diseases of all kinds [87,88,89,90,91,92,93]. Research on the biology of stress shows that being exposed to “toxic” levels of stress during early life harms the developing brain and other organs. Toxic stress occurs when a child experiences strong, frequent or prolonged adversity, such as economic hardship, abuse or exposure to violence, substance abuse, mental illness and parental divorce. An estimated 50% of the population in Europe has experienced at least one ACE which makes it an important public health concern [94]. Countries that do better for children often do better for adults, but well-being outcomes for these two groups are not always well-aligned [95]. This implies that these countries need to do better for their children if they are to maintain the levels of well-being enjoyed by today’s adults over time.

## Conclusion

The components that determine success of a national integrated care programme are multifactorial in nature and are characterized by a complex interplay. The Policy Guide on Integrated Care (PGIC) provides an insight and support to what policy makers can do at both the national and international level to improve integrated care for people with chronic conditions. Based on the findings from the FP-7 EU Project Integrate we argue that a comprehensive systems perspective should guide the development of integrated care towards better health practices, education, research and policy.
